# Toxic Effects of Urea

**Published:** 1917-04

**Authors:** 


### ABSTRACTS

Have you ever felt that this department of the BUFFALO MEDICAL JOURNAL was lacking in abstracts of your specialty, or that such as were published lacked the expert judgement which you yourself could have exercised in selection and preparation? If not, you are more charitable than the editor has been to himself. If so, will you assist in abstracting from a few journals, and thus make this department more representative and more valuable? Incidentally, there are few forms of study more helpful to the worker than this.

---

**Toxic Effects of Urea.** A. W. Hewlett, Q .O. Gilbert and A.

D. Wickett, Arch. of Int. Med., Nov. 1916, in 5 experiments, gave or took 100 grams of urea, either at once or divided over 3-6 hours, thus increasing the blood content from the normal of 4: 10,000 to 16-24.5: 10,000. Headache, dizziness, apathy, drowsiness, fatigue resulted, as in asthenic uraemia. Every gram of excess urea corresponds to a rise of about 25 m.g. per liter (or, according to the usual estimate of a total of 5 liters of blood, to an absolute amount of 1/8 gram in circulation.) They point out that the rise of nitrogenous substances in the blood, characteristic of the later stages of nephritis, urea shows the greatest increase. (However, we have shown that the ordinary tests for urea certainly include uric acid and probably most if not all other non-proteid forms of nitrogen, that it is, in short, an approximate method of determining the total non-proteid nitrogen. Whether specific tests for urea were carried out, we do not know). This report is interesting as tending to corroborate the early, crude conceptions of uraemia and to show that urea is not an entirely harmless substance.

